# Neurological Sequela of Acute Pesticide Poisoning Among Adults in Central Taiwan

**DOI:** 10.3389/fneur.2021.745265

**Published:** 2021-12-10

**Authors:** Yen-Chung Chen, Chin-Hsien Lin, Shey-Lin Wu

**Affiliations:** ^1^Department of Neurology, Changhua Christian Hospital, Changhua, Taiwan; ^2^Department of Neurology, National Taiwan University Hospital, Taipei, Taiwan

**Keywords:** neurological sequela, mortality, basal ganglion lesions on brain images, insecticide resistance action committee mode of action (MoA) classification, acute pesticide poisoning

## Abstract

**Background and Purpose:** Cases of acute pesticide poisoning account for significant morbidity and mortality in developing countries; however, its burden in Taiwan remains unknown. The study examined acute pesticide poisoning (APP) involving adults in the central region of Taiwan, which is a mainly agricultural sub-urban area.

**Methods:** The retrospective study evaluated the outcome and neurological sequelae of patients with APP in a Taiwanese cohort between April 2002 and February 2019. The pesticides were classified according to the Insecticide Resistance Action Committee Mode of Action (MoA) classification. The clinical characteristics, duration of hospitalization (days), follow-up duration (years), in-hospital mortality, neurological sequela, and imaging findings were recorded. Furthermore, multivariate logistic regression analyses were performed.

**Results:** We identified 299 patients with APP comprising 206 (68.9%) adult men with a mean exposure age of 56.4 ± 16.8 years. Paraquat, organophosphates, pyrethroids, carmabates, and phosphinic acid were the most commonly known reported poisoning agents. The mortality rate was highest in users with paraquat (77.1%), followed by phosphinic acid (22.2%), carbamates (16.7%), and organophosphates (15.8%). After a mean follows up of 3.69 ± 2.26 years, the most common neurological sequela was a cognitive decline (56 among 225 survivors, 24.89%), peripheral neuropathy (11 among 225 survivors, 4.89%), tremor (10 among 225 survivors, 4.44%), ataxia (3/225, 1.33%), and parkinsonism feature (2/225, 0.89%). Brain imaging studies revealed basal ganglion lesions on CT or hyperintensity on T2-weighted MRI images in 26 among 46 patients (56.5%). The basal ganglion lesions on brain imaging had a positive correlation with neurological sequelae.

**Conclusion:** Acute pesticide poisoning (APP)-related mortality is high especially paraquat intoxication, and cognitive decline, as well as peripheral neuropathy, were the most common neurological sequelae among survivors, which is highly correlated with basal ganglia lesions on brain imaging.

## Introduction

Acute pesticide poisoning is a global health concern with acute manifestations of cardiorespiratory, central nervous system, and gastrointestinal illness (1). Pesticides cause an estimated 200,000 acute poisoning deaths each year, 99% of which occur in developing countries (2). According to the report of the United Nations, pesticide poisoning inflicts substantial costs on Governments and has catastrophic impacts on the environment, human health, and society (3). Even people who survived a single acute pesticide poisoning (APP) event could have potential psychological and neurological deficits after years (4, 5). More and more studies have demonstrated that exposure to hazardous pesticides is strongly associated with growth problems, endocrine disruption, malignancy, nervous system disorders (especially neuropathy and neurodegenerative diseases like Alzheimer's and Parkinson's diseases), and sterility (6–16).

For nervous system sequelae, basal ganglia are vulnerable to toxic damage in the absence of a sufficient detoxification pathway, so brain imaging findings on computed tomography or magnetic resonance imaging are helpful tools for prognostic evaluation and possible pathophysiology studies based on different localizations (17–19).

Since pesticides are functionally divided into various types with different mechanisms such as insecticides, disinfectants, herbicides, fumigants, repellents or combined, they could have diverse acute symptoms and long-term impacts on exposed people (20). However, the different impacts on neurological sequelae and brain imaging presentations between different types of APP are limited.

We aimed to investigate the burden (especially mortality), chronic neurological sequela, and brain imaging patterns associated with the different subtypes of APP involving adults in the central region of Taiwan, which is a major agricultural sub-urban county.

## Methods

### Data Source

This retrospective cohort study was conducted at a poison center of a tertiary care hospital located in an important agricultural district in central Taiwan and received institutional review board approval from the Changhua Christian Hospital committee for the Human Subjects Protection (200316).

**Figure 1:**
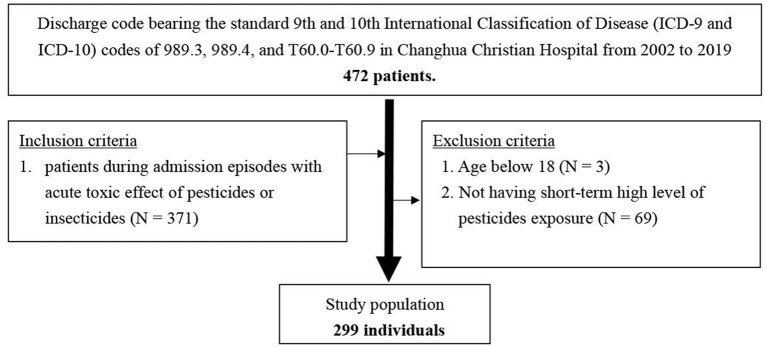
Flowchart of study samples selection from Changhua Christian Hospital.

Consecutive hospital admissions of patients ≥18 years old from April 2002 to February 2019 with a discharge code bearing the standard 9th and 10th International Classification of Disease (ICD-9 and ICD-10) codes of 989.3, 989.4, and T60.0–T60.9, and admission episodes with a confirmed diagnosis of the acute toxic effect of pesticides or insecticides were included. We excluded patients younger than 18 years and those that did not have short-term high-level pesticide exposure (Figure 1).

Demographic and comorbidity data, including age at admission, gender, exposure substances classification, underlying diseases such as metabolic diseases, cardiovascular diseases, malignancy, and neuropsychological diseases were retrieved. The pesticides were classified according to the Insecticide Resistance Action Committee Mode of Action (MoA) classification (21).

### Outcomes Measures

The major outcome was in-hospital mortality. Minor outcomes included neurological sequelae and basal ganglion lesions on CT or hyperintensity on T2-weighted MRI images. The neurological sequela was screened through a basic neurological examination at the office and compared with previous medical records to confirm whether they occurred after this episode of acute poisoning. For cognitive declination, we diagnosed based on clinical visiting data whether the patients' have an impaired ability to acquire and remember new information or impaired reasoning and handling of complex tasks, poor judgment or impaired visuospatial abilities or impaired language functions or changes in personality, behavior, or comportment. The clinical characteristics record the duration of hospitalization (days), follow-up duration (years), ICU admission for ventilator support, mortality, neurological sequela, and imaging findings.

### Data Analysis

Baseline characteristics were reported using frequencies and proportions for categorical variables and using mean values and standard deviations for continuous variables. A multiple logistic regression model was developed to determine the factors associated with neurological sequelae. The model included exposure age, gender, admission to ICU for ventilator support or not, basal ganglion lesions on brain imaging, and the most common poisoned pesticides and insecticides.

Overall survival for all groups (different pesticides or insecticides) was defined from the time of admission to the time of death or discharge. Survival time was censored for patients alive at the end of the discharge day. Estimates and 95% CIs of overall survival proportions were computed using the Cox proportional hazards regression, and survival distributions were compared across groups using the log-rank test.

A multivariate Cox proportional hazards model was developed for all patients who were poisoned to predict factors associated with survival, shown as hazard ratios (HRs) with 95% CIs. The model was adjusted for exposure age, present basal ganglion lesions on brain imaging, sex, and pesticides subgroups. Two-sided statistical tests were specified in all analyses and *p* < 0.05 were considered statistically significant. Statistical analysis was conducted using the procedures available in the licensed MedCalc Statistical Software version 19.5.3.

## Results

**Figure 2:**
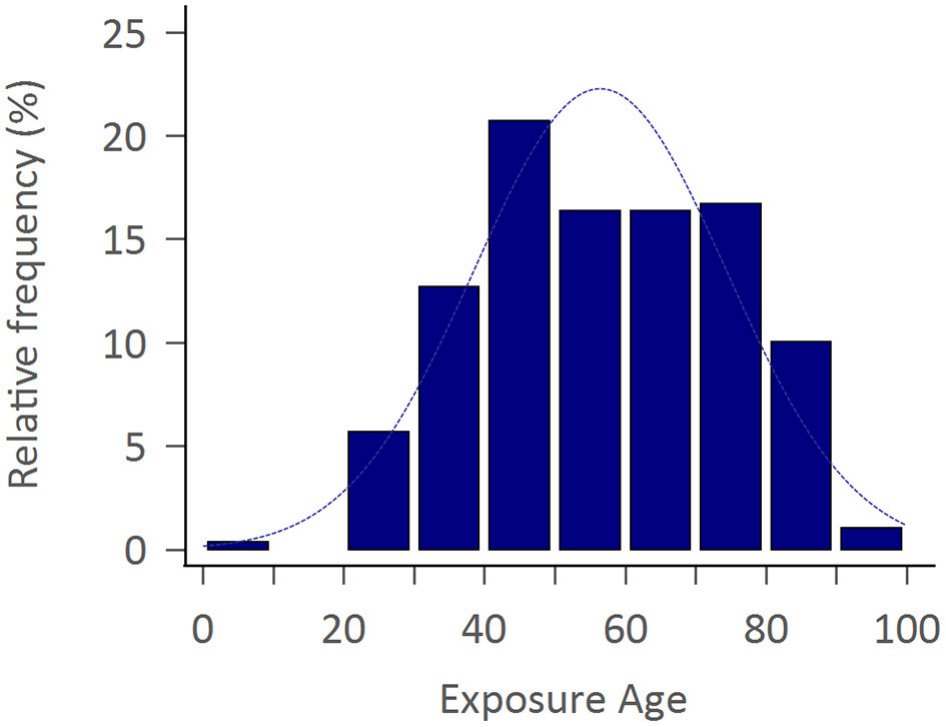
Exposure age distribution.

**Figure 3:**
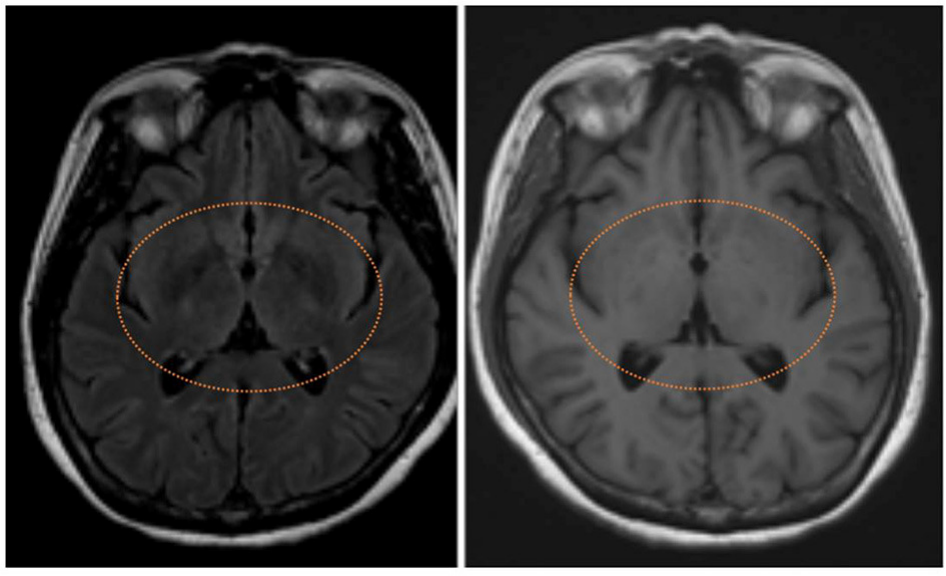
T2 fluid-attenuated inversion recovery magnetic resonance image showing symmetrical hyperintensities in bilateral basal ganglion (left) with T1 hypointensities (right) of neonicotinoids intoxication patient.

The study consisted of 299 APP cases of whom 206 (68.9%) were adult men with the mean exposure age of 56.4 ± 16.8 years. Figure 2 shows the distribution of exposure age. Paraquat, organophosphates, pyrethroids, carbamates, and phosphinic acid were the most common amongst known reported poisoning agents. The mortality rate was highest with paraquat users (77.1%), followed by phosphinic acid users (22.2%), carbamates users (16.7%), and organophosphates users (15.8%) (Table 1). After a mean follows up of 3.69 ± 2.26 years, the most common neurological sequelae were cognitive decline (56 among 225 survivors, 24.89%), peripheral neuropathy (11 among 225 survivors, 4.89%), tremor (10 among 225 survivors, 4.44%), ataxia (3/225, 1.33%) and parkinsonism feature (2/225, 0.89%). Brain imaging studies revealed basal ganglion lesions on CT or T2 weighted MRI images in 26 among 46 patients (56.5%) (Figure 3). All patients' features, distinct by subgroup, are listed in Table 1. For peripheral neuropathy, 6 of the 11 patients were diagnosed with sensorimotor polyneuropathy through nerve conduction examination. Others were diagnosed clinically by neurologists. All patients with tremors presented with action tremors in their hands. The two Parkinsonism patients with bradykinesia under different pesticide poisoning poorly responded to dopaminergic therapy. Subgroups with fewer than 3 are not listed in the table.

**Table 1:** Clinical characteristics, prognosis and neurological sequela of patients in this study.

	**AChE inhibitors: Organophosphate *N* = 57**	**AChE inhibitors: Carbamates *N* = 6**	**Sodium channel modulators: pyrethrins, pyrethroids *N* = 16**	**Photosystem I-electron diversion/ bipyridylium/ paraquat *N* = 70**	**Inhibition of EPSP synthase/ glycine *N* = 13**	**Inhibition of DHP *N* = 7**	**Inhibition of glutamine synthetase/ phosphinic acid *N* = 9**
Current age	63.0 ± 17.1	63.3 ± 23.8	74.9 ± 14.1	59.7 ± 18.7	59.0 ± 20.5	59.5 ± 20.0	65.7 ± 13.8
Age at exposure	57.1 ± 17.2	57.8 ± 22.6	68.9 ± 14.9	53.9 ± 18.6	54.2 ± 20.7	52.8 ± 20.2	60.7 ± 14.8
Males	40 (70.2%)	6 (100%)	7 (43.7%)	54 (81.4%)	9 (69.2%)	5 (71.4%)	6 (66.7)
Hospitalization days	5.6 ± 3.2	4.6 ± 3.5	4.7 ± 4.2	4.4 ± 2.9	4.9 ± 3.0	1.7 ± 0.7	3.3 ± 2.8
ICU stay	20 (35.1%)	2 (33.3%)	2 (12.5)	31 (44.3%)	4 (30.7%)	0 (0)	3 (33.3%)
F/U (years)	3.8 ± 2.1	4.6 ± 2.8	3.7 ± 2.3	3.8 ± 1.7	3.3 ± 1.5	3.1 ± 2.8	4.6 ± 2.8
Mortality	9 (15.8%)	1 (16.7%)	1 (6.3%)	54 (77.1%)	1 (7.7%)	1 (14.3%)	2 (22.2%)
mRS for those who survived	2.1 ± 1.8	1.7 ± 1.4	2.6 ± 2.2	3.9 ± 2.1	2.0 ± 1.5	2.1 ± 1.8	2.4 ± 2.1
**Neurological sequela**							
Neuropathy	3/46 (6.5%)	1/6 (16.7%)	1/14 (7.1%)	0	3/7 (42.8%)	0	3/5 (60%)
Tremor	1/46 (2.2%)	2/6 (33.3%)	2/14 (14.3%)	0	0	1/2 (50%)	0
Cognitive decline	13/46 (28.3%)	3/6 (50%)	3/14 (21.4%)	3/7 (42.8%)	4/7 (57.1%)	2/2 (100%)	2/5 (40%)
Others	1/46 (2.2%): epilepsy	0	0	0	0	0	0
**Brain image**							
BG lesions	9/12 (75.0%)	5/5 (100%)	1/5 (20%)	7/8 (87.5%)	2/4 (50%)	N/A	1/2 (50%)

*EPSP, 5-enolpyruvylshi kimate-3phosphate; DHP, dihydropteroate synthase; AChE, Acetylcholinesterase*.

Multivariate logistic regression analysis showed that only present basal ganglion lesions on brain imaging (odds ratio [OR]: 4.16, 95% CI: 1.48–11.74, *p* = 0.007) were found to be significantly associated with neurological sequelae (Table 2).

**Table 2:** Multiple logistic regression analysis of risk factors associated with neurological sequela.

**Variable**	**Odds ratio**	**95% CI**	***p*-value**
Exposure age	1.00	0.98–1.02	0.86
Admission to ICU for ventilator support	1.70	0.63–4.61	0.30
Basal ganglion lesions on brain image	4.16	1.48–11.74	<0.01^*^
**Gender**			
Female	Reference	-	-
Male	1.17	0.54–2.57	0.68
**Pesticides**			
AChE inhibitors: Organophosphate	0.68	0.16–3.00	0.61
Sodium channel modulators: pyrethrins, pyrethroids	0.51	0.06–4.42	0.54
Photosystem I-electron diversion/bipyridylium/paraquat	0.46	0.07–3.14	0.43
Inhibition of EPSP synthase/glycine	2.44	0.41–14.52	0.33
Inhibition of DHP	4.49	0.58–34.70	0.15
AChE inhibitors: Carbamates	0.55	0.04–8.16	0.66
Inhibition of glutamine synthetase/phosphinic acid	1.82	0.21–15.39	0.58

* *p <0.05*.

**Figure 4:**
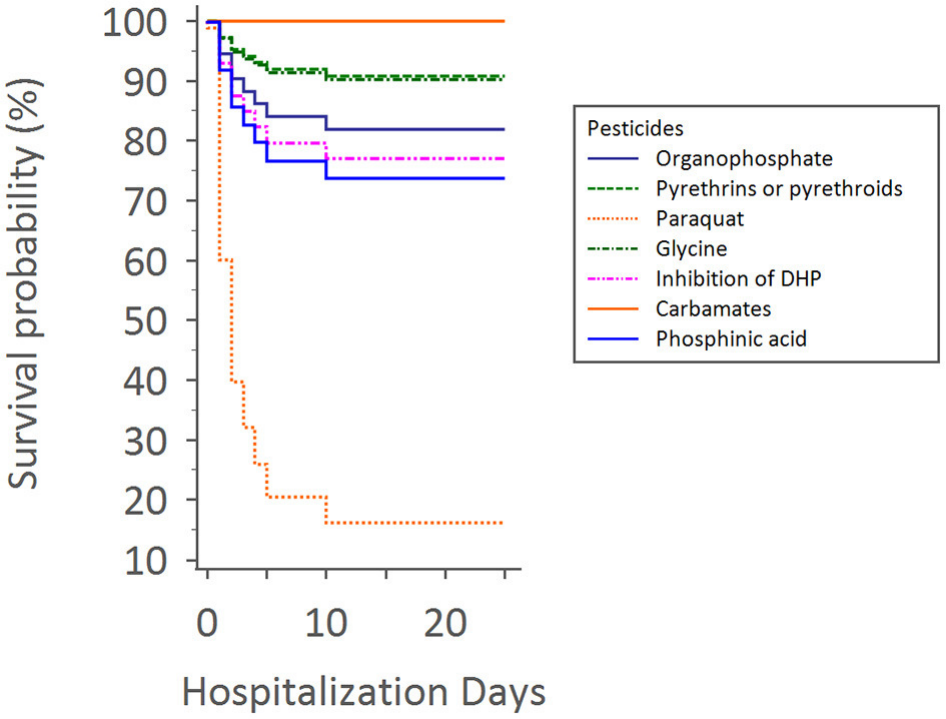
Cox regression for survival analysis during hospitalization times with different pesticides.

Upon Cox regression for survival analysis during hospitalization, paraquat poisoning was confirmed as an independent predictor (*p* < 0.0001) (Figure 4). After adjustment, we found that advanced exposure age (OR 1.03, 95% CI 1.01–1.04, *p* = 0.0001) was significantly associated with mortality (Table 3).

**Table 3:** Cox proportional-hazard analysis of mortality.

**Risk factor**	**Reg. Coeff**.	**Hazard ratio (95% CI)**	***p*-value**
**Pesticides**			
AChE inhibitors: Organophosphate	Reference	-	-
Sodium channel modulators: pyrethrins, pyrethroids	−0.66	0.51 (0.06– 4.17)	0.533
Photosystem I-electron diversion/bipyridylium/paraquat	2.11	8.22 (3.97–16.93)	<0.001^*^
Inhibition of EPSP synthase/glycine	−0.64	0.52 (0.07–4.15)	0.541
Inhibition of DHP	0.37	1.45 (0.18–11.52)	0.727
AChE inhibitors: Carbamates	−11.31	0.00	0.961
Inhibition of glutamine synthetase/phosphinic acid	0.46	1.59 (0.34–7.37)	0.553
Exposure age	0.02	1.02 (1.01–1.03)	0.002^*^
Gender (male vs. female)	0.04	1.04 (0.59–1.82)	0.882

* *p <0.05*.

*EPSP, 5-enolpyruvylshi kimate-3phosphate; DHP, dihydropteroate synthase; AChE, Acetylcholinesterase*.

## Discussion

This retrospective study showed that patients with acute pesticide poisoning have a high associated mortality rate, especially when the pesticide involved is paraquat; also, most people with neurological sequelae who survived APP had cognitive function impairment as well as peripheral neuropathy. Bilateral basal ganglion signal abnormalities' involvement on CT or MRI was positively correlated with neurological sequelae.

Earlier nationwide acute pesticide poisoning research in Taiwan mostly focus on organophosphate pesticides (22). Our study estimated the burden of acute poisoning by different pesticides or insecticides in the suburban of central Taiwan. From our study, most exposures were acute ingestion with organophosphate pesticide, which is an agent of acetylcholinesterase inhibitor to attempt suicide, and our result is compatible with global systemic review data (23). Males were more numerous (68.9%) in our study. Most published pesticide exposures in Asia involve acute ingestions in male adults' intent on self-harm (22, 24). Acute intoxication by pesticides or insecticides or herbicides causes neurotoxicity mostly by inhibiting the normal functioning of neurotransmitters or inducing oxidative stress; however, most of their effects are minor to moderate effects (25, 26). We followed up these patients for chronic neurological sequelae and found that cognitive function impairment and peripheral neuropathy constitute the majority of these sequelae, which is in line with the findings of previous studies (25). More than half of our patients with chronic neurological sequela were poisoned by acetylcholinesterase inhibitors. Nevertheless, significant morbidity and mortality rates were recorded in over three-quarters of all paraquat exposed areas from our investigation. Indika et al. suggested that acute paraquat poisoning could lead to multi-organ failure within a couple of hours to days, causing extremely high mortality rates (27).

The mortality rates reported in this study are similar to the previously reported acetylcholinesterase inhibitor poisoning mortality rate of 10–20% in many Asian and developing countries throughout the world (22, 28), and the rates of survival from paraquat poisoning are as low, as previous similar studies have reported (29).

The basal ganglia are a bunch of nuclei in the subcortical area that are responsible for crucial functions such as motor control and learning, executive function, emotion, and behaviors. A large number of robust pieces of evidence show that basal ganglia are vulnerable to toxic, metabolic, or ischemic damage; so, many studies show that bilateral basal ganglion image signal abnormalities are seen after intoxication, including acute pesticides poisoning (18, 30). From our study, it can be seen that basal ganglion lesions on brain imaging have a positive correlation with neurological sequelae which is compatible with earlier studies.

The main strength of this study is its detailed classification of pesticides using real-world data. However, the result should be interpreted in the context of the following limitations. First, it is a retrospective study without a normal control group, and the diagnosis of APP in this study relied mostly on administrative diagnosis codes. Not every patient was diagnosed directly through a neurologist. However, we had traced back every single patient's medical record to confirm which kind of pesticide they were exposed to, and the diagnosis correlated with the patient's present illness, and we also reviewed all the medical records case by case before the exposure to make sure the sequelae are not relevant to their earlier illness. Second, although we adjusted for as much bias as we could by using multivariable analysis to balance the baseline differences between the groups and eliminate residual confounding effects, biases related to unmeasured confounders remain a potential issue given the nature of this study. Third, patients may not have been followed up at a neurology clinic; so, subtle or mild neurological problems may have been underdiagnosed. Finally, this was a single-institution study, which means there might have been selection bias. Nevertheless, our burden of the neurological sequelae or mortality is consistent with previous studies in Asia even worldwide.

In conclusion, the mortality rate associated with acute pesticide intoxication is high and strongly correlated with paraquat. Bilateral basal ganglion signal abnormalities findings on imaging are significantly associated with neurological sequelae. Cognitive function impairment and peripheral neuropathy are the most commonly encountered residual neurological symptoms. Our results shed light on the burden and influences of acute pesticide poisoning. Further public health regulations should be put in place.

## Data Availability Statement

The original contributions presented in the study are included in the article/supplementary material, further inquiries can be directed to the corresponding author/s.

## Ethics Statement

The studies involving human participants were reviewed and approved by Changhua Christian Hospital Committee for the Human Subjects Protection (200316). The patients/participants provided their written informed consent to participate in this study.

## Author Contributions

S-LW contributed to the conception and design of the study. Y-CC organized the database and wrote the first draft of the manuscript. Y-CC and C-HL performed the statistical analysis. All authors contributed to manuscript revision, read, and approved the submitted version.

## Conflict of Interest

The authors declare that the research was conducted in the absence of any commercial or financial relationships that could be construed as a potential conflict of interest.

## Publisher's Note

All claims expressed in this article are solely those of the authors and do not necessarily represent those of their affiliated organizations, or those of the publisher, the editors and the reviewers. Any product that may be evaluated in this article, or claim that may be made by its manufacturer, is not guaranteed or endorsed by the publisher.
